# Alimentary Limb Obstruction Following One Anastomosis Gastric Bypass Due to a V-loc™ Suture

**DOI:** 10.7759/cureus.104483

**Published:** 2026-03-01

**Authors:** Amir Obeid, Miriam Obeid, Amir Farah, Sa'd Sayida

**Affiliations:** 1 General Surgery, HaEmek Medical Center, Afula, ISR; 2 General Surgery, Rambam Medical Center, Haifa, ISR; 3 Surgery, Medical College of Wisconsin, Division of Trauma and Acute Care Surgery, Milwaukee, USA; 4 General Surgery, Minimally Invasive and Bariatric Surgery Unit, Rambam Medical Center, Haifa, ISR

**Keywords:** alimentary limb obstruction, bariatric surgery, internal hernia, mini gastric bypass, one anastomosis gastric bypass, small bowel obstruction, v-loc™ suture

## Abstract

This case highlights a rare postoperative complication after one anastomosis gastric bypass (OAGB) in a bariatric patient. A middle-aged adult woman presented with recurrent abdominal symptoms due to mechanical obstruction of the alimentary limb caused by a V-Loc™ suture (Medtronic plc, Dublin, Ireland). Despite prior inconclusive imaging and endoscopy, diagnostic laparoscopy identified the obstruction. The patient recovered well postoperatively. This case underscores the importance of clinical vigilance and awareness of potential complications associated with surgical sutures in bariatric procedures.

## Introduction

Advances in surgical techniques and technology, especially in bariatric surgery, are an ongoing process that has enabled surgeons to achieve operative success and satisfaction for morbidly obese patients undergoing these procedures. Although the incidence of complications remains an issue for many surgeons and may have postoperative consequences, abdominal symptoms following laparoscopic bariatric surgery remain a challenge for the surgeon in obtaining a definite diagnosis. There are various complications that can lead to postoperative abdominal pain and cause gastrointestinal impairment, for example, bowel obstruction, which has been seen following Roux-en-Y gastric bypass [1].

Here, we report a rare case of bowel obstruction of the alimentary limb following one anastomosis gastric bypass (OAGB) as a result of a V-Loc™ suture (Medtronic plc, Dublin, Ireland) that grasped and obstructed the alimentary limb, causing obstruction and chronic abdominal complaints. After a literature review, to date, only descriptions of obstruction following Roux-en-Y gastric bypass have been reported, which verifies its incidence in other bariatric procedures. This case report describes a middle-aged adult woman who had a mini gastric bypass and cholecystectomy in the past and presented to the emergency department with recurrent complaints of abdominal pain and vomiting. The patient had normal CT and gastroscopy findings in the past. However, on her current admission, she had worsening symptoms and abdominal tenderness, leading to the discovery of an internal hernia causing small bowel obstruction. As a result of our case, we performed a literature review of similar reported cases that used V-Loc™ sutures during bariatric-metabolic surgery and compared the outcomes.

## Case presentation

The patient is a middle-aged adult female with a history of morbid obesity, for which she underwent a laparoscopic OAGB (also known as mini gastric bypass) two years prior to presentation. Additionally, she had undergone a laparoscopic cholecystectomy one year before her current admission. Over the preceding months, she had presented on multiple occasions to the emergency department with episodic, nonspecific abdominal pain and intermittent vomiting, which were variably attributed to functional gastrointestinal complaints given repeated unremarkable evaluations, including abdominal computed tomography (CT) scans and upper endoscopy.

At this presentation, the patient reported several days of progressively worsening epigastric and periumbilical abdominal pain associated with nausea and bilious vomiting. She denied fever, chills, jaundice, or changes in bowel habits. On physical examination, she was hemodynamically stable, afebrile, and well perfused. Abdominal examination revealed mild to moderate diffuse tenderness, most pronounced in the upper quadrants, without signs of peritonitis, guarding, or rebound tenderness. Her bowel sounds were present but hypoactive.

All laboratory investigations, as presented in Table 1, including complete blood count (white blood cell count 8.5), serum electrolytes, liver function tests, amylase, lipase, and arterial blood gas analysis, were within normal limits.

**Table 1:** Laboratory findings and normal values

Parameter	Result	Reference Range	Units
White blood cell count	8.5	4.0-11.0	×10⁹/L
Hemoglobin	14.2	13.5-17.5	g/dL
Platelet count	245	150-400	×10⁹/L
Sodium (Na⁺)	139	135-145	mmol/L
Potassium (K⁺)	4.2	3.5-5.0	mmol/L
Chloride (Cl⁻)	102	98-106	mmol/L
Bicarbonate (HCO₃⁻)	24	22-28	mmol/L
AST	22	10-40	U/L
ALT	25	7-56	U/L
Alkaline phosphatase (ALP)	78	44-147	U/L
Total bilirubin	0.8	0.3-1.2	mg/dL
Amylase	65	30-110	U/L
Lipase	42	0-60	U/L
Arterial pH	7.4	7.35-7.45	
PaCO₂	40	35-45	mmHg
HCO₃⁻ (ABG)	24	22-26	mmol/L

**Figure 1:**
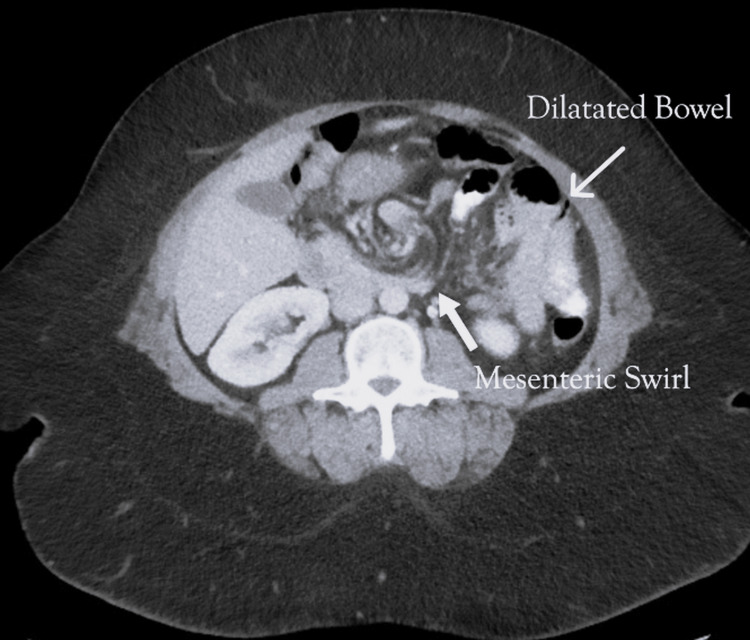
Computed tomography imaging of mesenteric swirling and proximal bowel dilatation.

This provided no overt indication of acute inflammatory or ischemic pathology. Given the persistent nature of her symptoms and her history of bariatric surgery, a contrast-enhanced CT scan of the abdomen and pelvis was performed. Imaging revealed subtle findings concerning for internal herniation, including swirling of mesenteric vessels and proximal small bowel dilation suggestive of partial obstruction (Figure 1). 

**Figure 2:**
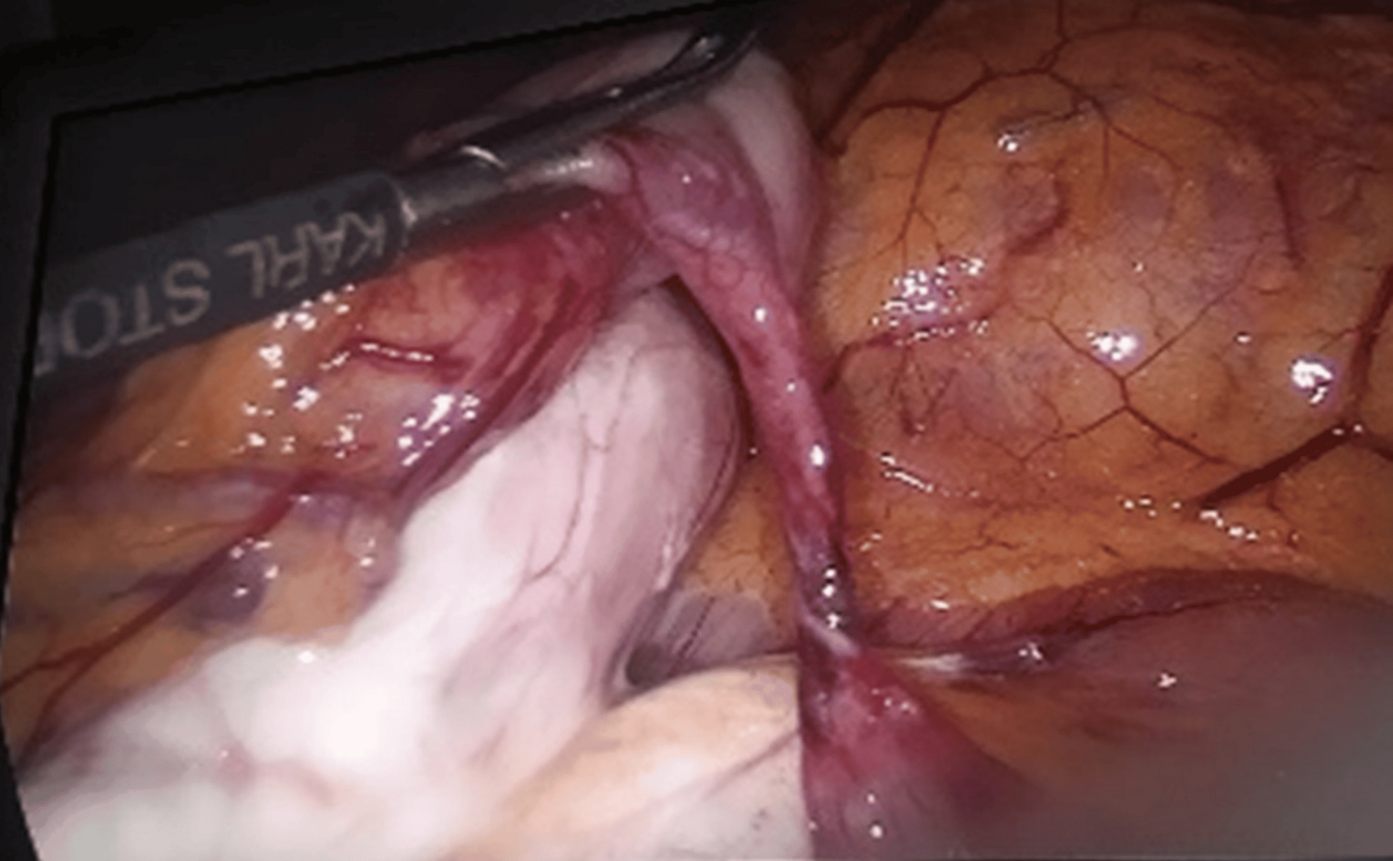
Intraoperative laparoscopic view demonstrating the obstructed alimentary limb following a mini gastric bypass procedure. The obstruction is caused by entrapment from a V-loc™ suture (Medtronic plc, Dublin, Ireland) encircling the limb.

**Figure 3:**
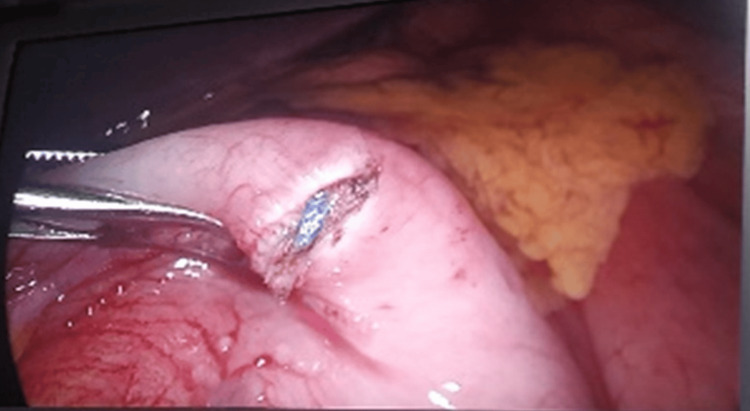
Close-up intraoperative view showing the V-loc™ suture (Medtronic plc, Dublin, Ireland) grasping and compressing the alimentary limb, contributing to the mechanical obstruction and symptomatic presentation.

**Figure 4:**
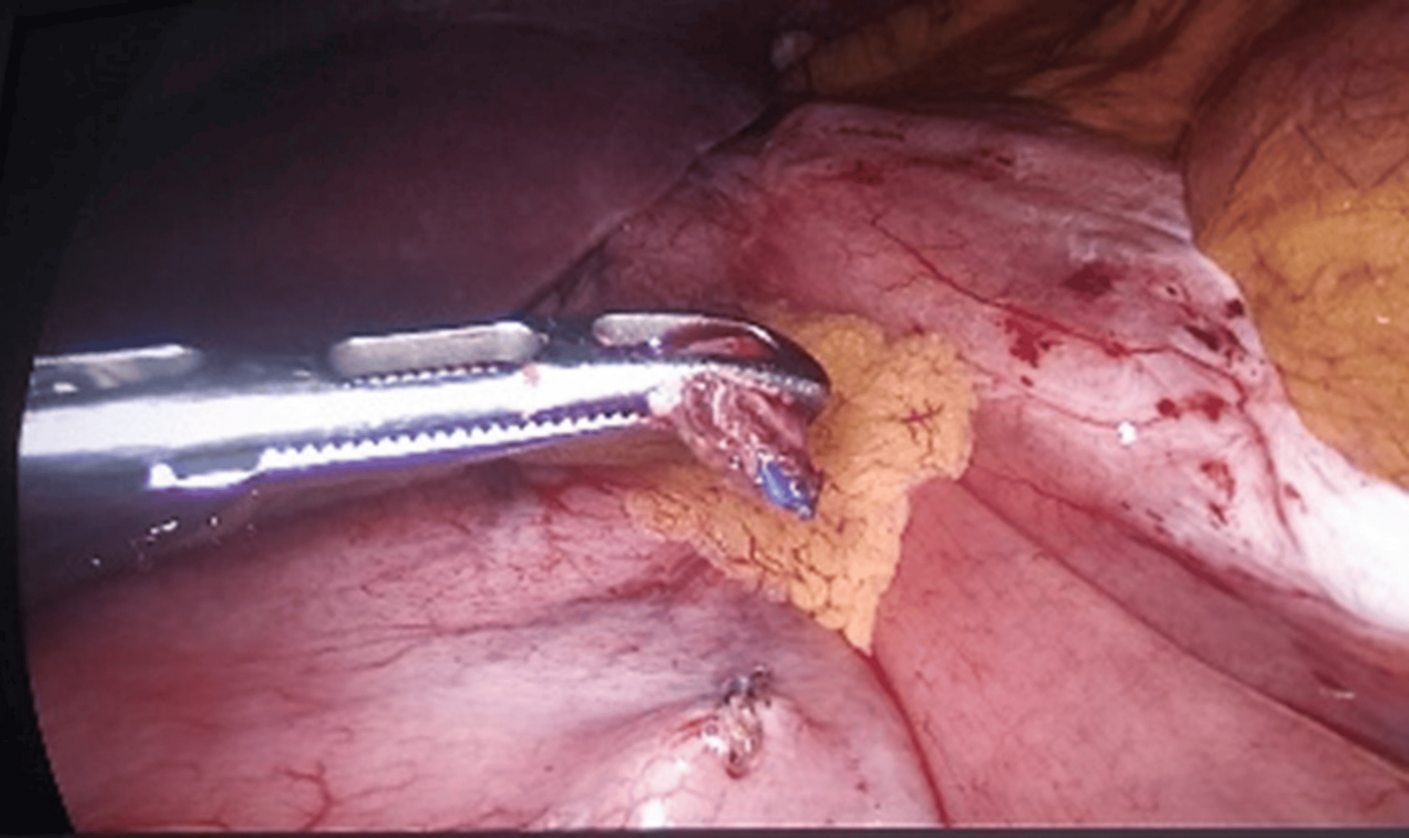
Laparoscopic image taken during adhesiolysis, illustrating the moment of V-loc™ suture (Medtronic plc, Dublin, Ireland) release, effectively resolving the alimentary limb obstruction.

Given the concerning radiographic findings and the clinical context of recurrent, unexplained abdominal pain in a post-OAGB patient, the decision was made to proceed with diagnostic laparoscopy. Intraoperatively, an obstructive band was identified involving a V-Loc™ barbed suture that had inadvertently ensnared a segment of the alimentary (efferent) limb of the bypassed small bowel, creating a fixed point of obstruction (Figures 2-4).

Additionally, there were dense adhesions in the vicinity of the gastrojejunostomy site contributing to partial luminal compromise. The obstructing suture was carefully dissected and divided, and adhesiolysis was performed to relieve the obstruction.

No evidence of bowel ischemia, perforation, or other intra-abdominal complications was observed. The procedure was concluded uneventfully. The patient recovered well postoperatively, was able to tolerate oral intake by postoperative day one, and was discharged home in stable condition on postoperative day two.

At follow-up in the outpatient clinic, the patient reported complete resolution of her prior symptoms, with no recurrence of abdominal pain, nausea, or vomiting, suggesting definitive resolution of the mechanical obstruction.

## Discussion

Internal hernias are an uncommon yet potentially life-threatening complication following bariatric surgery, especially in patients who have undergone gastric bypass procedures. The reported incidence varies between 0.2% and 9%, depending on operative technique, whether mesenteric defects are closed, and the extent of postoperative weight loss [1]. These hernias most frequently occur due to mesenteric defects created during intestinal rerouting and are further exacerbated by the loss of mesenteric fat, which enlarges potential herniation spaces and increases bowel mobility within the abdominal cavity.

Common sites of internal herniation following gastric bypass include the transverse mesocolon defect in retrocolic Roux-en-Y reconstructions, Petersen’s space between the mesentery of the alimentary limb and the transverse colon, and the mesenteric defect at the jejunojejunostomy site [2]. In laparoscopic approaches, the likelihood of internal herniation is higher because fewer adhesions form postoperatively, allowing for greater small bowel mobility and increased risk of herniation.

Clinically, patients may present with nonspecific gastrointestinal complaints, including intermittent abdominal pain, nausea, vomiting, and symptoms suggestive of partial or complete bowel obstruction. Due to the vague nature of these symptoms, diagnosis is often delayed or missed entirely. CT imaging with oral and intravenous contrast remains the gold standard for diagnosing internal hernias, offering sensitivity and specificity rates of approximately 92% and 95%, respectively [3]. Radiological signs include the "mesenteric swirl," indicating twisted mesenteric vessels, clustered small bowel loops, and displacement of intestinal landmarks [4].

In the presented case, a young adult woman experienced multiple emergency department visits for recurrent abdominal pain and vomiting two years after undergoing mini gastric bypass (OAGB). Despite multiple imaging and endoscopic evaluations, no definitive pathology was identified until the patient underwent diagnostic laparoscopy. Intraoperatively, a rare mechanical cause of obstruction was found: a V-Loc™ barbed suture had entrapped a segment of the alimentary limb, causing partial obstruction and symptoms mimicking an internal hernia.

V-Loc™ sutures are barbed, self-anchoring sutures that eliminate the need for knot tying and facilitate even tissue approximation. They have gained popularity in minimally invasive surgery due to their efficiency and ease of use. However, concerns are emerging regarding their safety profile in gastrointestinal surgery, particularly in dynamic anatomical sites such as the mesentery or mobile loops of bowel. Several case reports and studies have implicated barbed sutures in causing adhesions, bowel entrapment, and mechanical obstruction in bariatric and other abdominal procedures.

Stenberg et al. demonstrated the importance of mesenteric defect closure in laparoscopic gastric bypass surgery, suggesting that failure to securely close these defects can result in a higher incidence of internal herniation [5]. Although their study did not focus solely on suture material, the implication was that the method and integrity of defect closure are critical determinants of patient outcomes. In a more focused study, Geubbels, Noëlle et al. evaluated internal herniation following Roux-en-Y gastric bypass and reported that barbed sutures, particularly V-Loc™, were associated with a significantly increased risk of internal hernia formation compared with conventional sutures [6].

The mechanism by which barbed sutures may lead to bowel entrapment or herniation includes the creation of a fixed point of tension, where a loop of bowel can become ensnared or compressed. Furthermore, the non-smooth surface of the barbed suture may encourage adhesion formation or local inflammation, setting the stage for delayed obstruction or stricture. In our case, the suture had inadvertently created a noose-like effect, tightly encircling the efferent limb, thereby mimicking the symptoms and radiological signs of an internal hernia.

This case highlights several important clinical lessons. First, in patients with a history of bariatric surgery, especially those who have undergone OAGB or Roux-en-Y procedures, recurrent or unexplained abdominal symptoms should prompt early consideration of internal hernias or mechanical complications. Second, while imaging studies are indispensable, they are not infallible. In cases where clinical suspicion remains high despite inconclusive imaging, diagnostic laparoscopy should be pursued to confirm or rule out critical intra-abdominal pathology.

Furthermore, this case underscores the importance of surgical material selection. While V-Loc™ sutures offer clear operative advantages, their use in mobile or dynamic tissue planes, particularly near anastomoses or mesenteric defects, should be approached with caution. It is advisable for surgeons to critically assess the placement and tension of barbed sutures, particularly when working in areas at high risk for entrapment or adhesion.

In conclusion, this case contributes to the growing recognition of rare yet serious complications associated with advanced surgical materials. As bariatric procedures continue to evolve and gain prevalence, so must our understanding of the nuanced risks they present. Awareness, timely surgical evaluation, and informed material selection remain the cornerstones of safe and effective postoperative care in the bariatric population.

## Conclusions

Recurrent abdominal symptoms following one anastomosis gastric bypass (OAGB) should prompt a comprehensive diagnostic approach, with careful consideration of uncommon mechanical etiologies. Although barbed sutures such as V-Loc™ are widely used and offer technical advantages, they may contribute to rare but clinically significant postoperative complications, including alimentary limb obstruction and internal herniation. In patients with persistent or unexplained symptoms despite inconclusive radiologic and endoscopic evaluation, early diagnostic laparoscopy remains a crucial tool for definitive diagnosis and timely management. Heightened awareness of such suture-related complications may facilitate earlier intervention and improve postoperative outcomes in bariatric surgery patients.

## References

[1] Aghajani E, Jacobsen HJ, Nergaard BJ, Hedenbro JL, Leifson BG, Gislason H (2012). Internal hernia after gastric bypass: a new and simplified technique for laparoscopic primary closure of the mesenteric defects. J Gastrointest Surg.

[2] Lanzetta MM, Masserelli A, Addeo G (2019). Internal hernias: a difficult diagnostic challenge. Review of CT signs and clinical findings. Acta Biomed.

[3] Lockhart ME, Tessler FN, Canon CL (2007). Internal hernia after gastric bypass: sensitivity and specificity of seven CT signs with surgical correlation and controls. AJR Am J Roentgenol.

[4] Blachar A, Federle MP, Pealer KM, Ikramuddin S, Schauer PR (2002). Gastrointestinal complications of laparoscopic Roux-en-Y gastric bypass surgery: clinical and imaging findings. Radiology.

[5] Stenberg E, Szabo E, Ågren G (2016). Closure of mesenteric defects in laparoscopic gastric bypass: a multicentre, randomised, parallel, open-label trial. Lancet.

[6] Geubbels N, Röell EA, Acherman YI, Bruin SC, van de Laar AW, de Brauw LM (2016). Internal herniation after laparoscopic Roux-en-Y gastric bypass surgery: pitfalls in diagnosing and the introduction of the Amsterdam classification. Obes Surg.

